# Electrochemical Surface Restructuring of Phosphorus-Doped Carbon@MoP Electrocatalysts for Hydrogen Evolution

**DOI:** 10.1007/s40820-021-00737-w

**Published:** 2021-10-21

**Authors:** Huimin Jiang, Liting Yan, Shuo Zhang, Yanchao Zhao, Xue Yang, Yameng Wang, Jianxing Shen, Xuebo Zhao, Lianzhou Wang

**Affiliations:** 1 School of Materials Science and Engineering, Qilu University of Technology (Shandong Academy of Sciences), No. 3501, Daxue Road, Changqing District, Jinan, 250353 People’s Republic of China; 2 Nanomaterials Centre, School of Chemical Engineering and Australian Institute for Bioengineering and Nanotechnology, The University of Queensland, St Lucia, QLD 4072 Australia; 3 College of Chemical Engineering, China University of Petroleum (East China), Huangdao District, No. 66, West Changjiang Road, Qingdao, 266580 People’s Republic of China

**Keywords:** Electrochemical surface restructuring, Hydrogen evolution, Molybdenum phosphide nanowires, Phosphorus-doped carbon, Synergistic interaction

## Abstract

The hydrogen evolution reaction (HER) through electrocatalysis is promising for the production of clean hydrogen fuel. However, designing the structure of catalysts, controlling their electronic properties, and manipulating their catalytic sites are a significant challenge in this field. Here, we propose an electrochemical surface restructuring strategy to design synergistically interactive phosphorus-doped carbon@MoP electrocatalysts for the HER. A simple electrochemical cycling method is developed to tune the thickness of the carbon layers that cover on MoP core, which significantly influences HER performance. Experimental investigations and theoretical calculations indicate that the inactive surface carbon layers can be removed through electrochemical cycling, leading to a close bond between the MoP and a few layers of coated graphene. The electrons donated by the MoP core enhance the adhesion and electronegativity of the carbon layers; the negatively charged carbon layers act as an active surface. The electrochemically induced optimization of the surface/interface electronic structures in the electrocatalysts significantly promotes the HER. Using this strategy endows the catalyst with excellent activity in terms of the HER in both acidic and alkaline environments (current density of 10 mA cm^−2^ at low overpotentials, of 68 mV in 0.5 M H_2_SO_4_ and 67 mV in 1.0 M KOH).

**Supplementary Information:**

The online version contains supplementary material available at 10.1007/s40820-021-00737-w.

## Highlights

- An electrochemical-induced surface restructuring strategy is developed to design phosphorus-doped carbon@MoP electrocatalysts which exhibits excellent activity for the hydrogen evolution reaction (HER) in both acidic and alkaline electrolytes.
- The activation process and the fundamental mechanism of the prominent synergistic interaction between the phosphorus-doped carbon and MoP are elucidated.

**Supplementary Information:**

The online version contains supplementary material available at 10.1007/s40820-021-00737-w.

## Graphical abstract

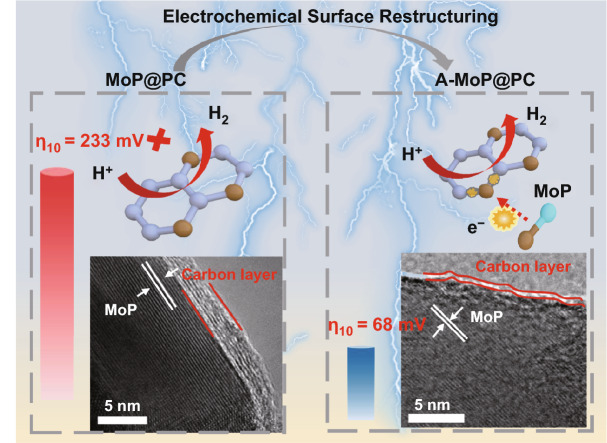

## Introduction

The development of advanced materials for renewable energy applications has attracted increasing research attention in recent years [1–4]. As an abundant, zero carbon emission source and renewable energy carrier, hydrogen plays an important role in sustainable energy and industrial chemical systems [5–8]. Hydrogen production through electrochemical water-splitting is a type of hydrogen-production technology that is advantageous owing to its high energy-conversion rate and environmentally friendly process [9–13]. Developing high-performance, low-cost electrocatalysts is an ideal method for solving the current energy crisis [14–18].

To date, transition-metal phosphides (TMPs) have already been widely used as active and low-cost hydrogen evolution reaction (HER) electrocatalysts [19–22]. Among the myriad existing TMPs, molybdenum phosphide (MoP) exhibits great potential for application owing to its intrinsic catalytic property [23–26]. However, the performances of TMPs cannot be fully maximized owing to the poor conductivity and the harsh conditions in strongly acidic or alkaline media, along with the fact that the high overpotential in electrocatalysis leads to corrosion, agglomeration, or oxidation. Therefore, maintaining the high stability of TMPs with the simultaneous enhancement of their activity under harsh conditions is a crucial challenge. Recently, encapsulating transition-metal nanoparticles with stable carbon layers has emerged as a novel strategy for designing active and durable catalysts [27, 28]. The ultrathin graphene shell significantly promotes electron transfer from the encapsulated metals to the graphene surface, which optimizes the electronic structure of the graphene surface and triggers the catalytic activity of the inert graphene surface. The synergistic effect of the metal core and carbon layer is strongly related to the thickness of the carbon layer. Density functional theory (DFT) calculations regarding the effect of the thickness of the carbon layer on its performance showed that the carbon shell surface was not likely to be activated if the layer number was greater than three [29]. However, it is difficult to control the thickness of the carbon layers, the unpredictable carbon layers that cover active species are often too thick, which could significantly reduce the catalytic activity.

Recent research on the activity of the HER and oxygen evolution reaction (OER) has suggested that the in situ reconstructed surface of catalysts under electrochemical conditions provides intrinsic active sites for high catalytic activities [30]. For example, Liu et al. discovered that phosphides derived from CoFeO@ black phosphorus (BP) formed during HER catalysis [31]. Driess et al. found that the surface reconstruction led to lattice vacancies and defects due to the loss of Li or Na in cobalt borophosphates during the HER [32]. However, owing to the poor structural flexibility of many materials, most surface reconstructions are triggered by electro-oxidation during the electrocatalysis process [33], and it is difficult to control the reconstruction during the HER. Furthermore, the evolution of the structural and physicochemical properties of catalysts in the electrocatalysis process makes it difficult to promote HER catalytic performance through electro-oxidation methods, especially when surface reconstruction occurs through water electrolysis. This is a significant bottleneck to tracking the structural evolution of catalysts and understanding the nature of catalytically active surfaces [34–36]. Further modulating the surface restructuring process and manipulating the in situ generated active surface species are even more challenging. Therefore, a controllable process is required to adjust the surface carbon structure of electrocatalysts and improve their catalytic properties [37–40].

**Fig. 1:**
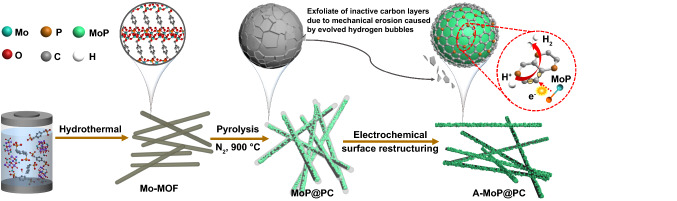
Schematic illustration for the synthesis of the A-MoP@PC

In this research, we report on the surface restructuring behavior of an electrocatalyst with MoP nanocrystals encapsulated in ultrathin phosphorus-doped carbon layers. A simple electrochemical cycling process is used to peel off the inactive carbon coating layer, which leads to the in situ generated active surface species boosting the HER catalytic activity of the electrocatalysts. The synthesis routine is schematically illustrated in Fig. 1. First, we developed a one-step strategy for the synthesis of a several-layer-thick phosphorus-doped carbon-coated MoP catalyst in a well-defined nanowire structure, derived from a phosphorous-containing Mo-Metal–organic framework (MOF) precursor without any additional phosphorus and carbon sources. Subsequently, the structural evolution of catalysts during a cyclic voltammetry (CV) process was applied to the electrocatalysts. It is important to note that these materials exhibit unusual surface restructuring behavior during the electrochemical process. Using simple low voltage electrochemical cycling in an acidic medium, the inactive surface carbon layers can be exfoliated, which makes the carbon layers thinner, allowing only 1–3 layers to be retained. Because of the high affinity between the MoP core and the P-doped carbon shell, a unique synergistic interaction at the interfaces boosts the electrocatalytic activities of the electrocatalyst. The charge-polarized P-doped carbon layer on the MoP surfaces exhibits excellent stability and catalytic activity. This practical surface restructuring strategy could be utilized to develop efficient electrochemical catalysts that are not only limited to use in the HER procedure.

## Experimental Section

### Synthesis of p-Xylylenediphosphonic Acid (H_4_xdp)

The ligand was synthesized by reacting alpha, alpha'-Dibromo-p-xylene with triethyl phosphite and followed by refluxing the obtained oil with conc. hydrochloric acid according to the literature method [41]. Block colorless crystals were obtained from the water solution by slow evaporation.

### Synthesis of [(MoO_2_)_2_(xdp)(H_2_O)_2_]·2H_2_O

Mo-MOF precursor was prepared according to previous work [41]. In a typical procedure, Na_2_MoO_4_·2H_2_O (0.240 g, 1.0 mmol) was stirred together with p-xylylenediphosphonic acid (H_4_xdp) (0.140 g, 0.5 mmol) in 16 mL deionized water. The pH of the solution was adjusted to 1 by dropwise addition of conc. hydrochloric acid. The acidified solution was then placed in a 25 cm^3^ Ace pressure tube and heated at 120 °C for 15 h. The resultant white crystalline material was thoroughly washed with deionized water several times and dried at 80 °C for 12 h under vacuum.

### Preparation of MoP@PC Nanowires

In a typical procedure, 500 mg Mo-MOF precursor was placed in a porcelain boat. Then, the boat was heated at 900 °C under a constant flow of N_2_ at 30 mL min^−1^ for 120 min with the warming rate of 20 °C min^−1^. The final black powder was collected when the temperature dropped to room temperature under N_2_.

### Electrochemical Activation

The *in situ* electrochemical activation was carried out in 0.5 M H_2_SO_4_ under a N_2_ atmosphere to avoid possible oxidation caused by O_2_ in air. This was conducted by using the three-electrode system of CHI 760E electrochemical workstation (CH Instruments, Inc., Shanghai). MoP@PC was used as the working electrode, carbon rod was used as the counter electrode, and Ag/AgCl (saturated KCl filled) was used as the reference electrode. The electrochemical activation was performed by cycle voltammetry (CV) from -0.2 to 0.2 V vs RHE in 0.5 M H_2_SO_4_.

### Characterization

The crystal structure of sample was characterized by powder X-ray diffraction (XRD) (PANalytical Inc.) using Cu Kα irradiation operating at 45 kV and 40 mA with a fixed slit. Morphology of sample was observed by a JEOL JSM-7500F (Japan) field-emission scanning electron microscopy (FESEM). High-resolution transmission electron microscopy (HRTEM) images were measured using a JEOL JEM2100F (Japan) transmission electron microscope. Nitrogen sorption isotherms were measured at 77 K using an Autosorb volumetric gas sorption analyzer (Quantachrome, USA). Thermogravimetric analyzer (TGA) was conducted on Mettler Toledo TGA/SDTA85. X-ray photoelectron spectroscopy (XPS) analyses were performed with a Thermo ESCALAB 250 (USA) spectrometer using an Al K_α_ (1486.6 eV) photon source. Raman spectrum was recorded using JY HR800 under ambient conditions. The X-ray absorption near-edge structure (XANES) measurement was taken at Singapore Synchrotron Light Source, facility for catalysis research (XAFCA) beamline.

Electrochemical measurements were taken at room temperature; catalyst ink was typically made by dispersing 20 mg of catalyst in 2 mL mixture of alcohol and water (3:1). After adding 0.5 mL of Nafion solution and ultrasonication, an aliquot of 5 µL was pipetted onto the glassy carbon electrode (0.0706 cm^2^) to reach the catalyst loading of 0.56 mg cm^−2^. In a three-electrode configuration, polarization curves were collected by CHI 760E electrochemical workstation at room temperature. Current density was normalized to the geometrical area of the working electrode. Polarization data are collected at the scan rate of 5 mV s^−1^. EISs were carried out in a potentiostatic mode in the frequency range of 10^6^ to 1 Hz with the amplitude of 5 mV.

## Results and Discussion

### Characterization of Morphology and Structure

**Fig. 2:**
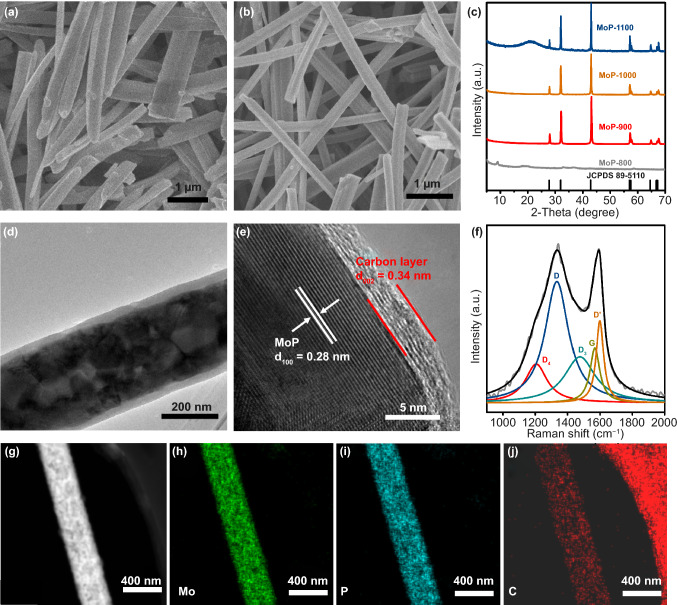
FESEM images of **a** Mo-MOF precursor and **b** MoP@PC. **c** XRD pattern of Mo-based MOF after being calcined at 700–1100 °C. **d** TEM and **e** HRTEM images of MoP@PC. **f** Raman spectra of MoP@PC. **g–j** TEM–EDS mapping of the MoP@PC

The MoP catalysts were synthesized via the one-step sintering of a Mo-based MOF. The XRD peaks of synthesized Mo-MOF agree well with the profile simulated from single-crystal structure data (Fig. S1) [41]. It can be seen in Fig. 2a that the Mo-based MOF has a regular morphology of nanowires, with diameters ranging from approximately 200 to 300 nm. Thermogravimetric (TG) analysis was carried out in N_2_ atmosphere to characterize the thermostability of the Mo-MOF precursor (Fig. S2). When the temperature is at around 135 °C, the sample lost about 12% of its weight including water, and small molecules adsorbed from air. The continuous weight-loss in the range of 800–940 °C corresponds to the decomposition of Mo-MOF precursor. The N_2_ adsorption/desorption isotherm at 77 K was performed to investigate specific surface area of Mo-MOF precursor. As shown in Fig. S3, the specific surface area of Mo-MOF precursor is 15.5 m^2^ g^−1^. Subsequently, the Mo-MOF precursor was heat-treated in a nitrogen atmosphere. Figure 2c shows the XRD pattern of the Mo-based MOF after being calcined at 800–1100 °C. It can be seen that the annealing temperature plays an important role in the fabrication process; the MoP phase was formed when the annealing temperature was higher than 900 °C. The diffraction peaks found through XRD at 27.8°, 31.9°, 42.8°, 56.9°, 57.4°, 64.5°, 66.8°, and 67.2° were ascribed to the (001), (100), (101), (110), (002), (111), (200), and (102) planes of hexagonal MoP (JCPDS No. 89-5110), respectively. Furthermore, no other peaks were observed, indicating the good crystallinity and purity of the MOF-derived MoP. The HER activity of these catalysts shown in Fig. S4 helped optimizing the annealing temperatures. The samples obtained at 900 °C (termed as MoP@PC) possessed better HER activity, which can be seen through the linear sweep voltammetry (LSV) curves in 0.5 M H_2_SO_4_, indicating that the annealing temperature of 900 °C is optimal for MoP formation and the electrocatalytic activity of MoP@PC. The morphological features of MoP@PC were obtained through field-emission scanning electron microscopy (FESEM) and TEM. The FESEM of MoP@PC (Fig. 2b) shows that the MoP@PC has a regular nanowire morphology, similar to that of the Mo-MOF precursor (Fig. 2a), indicating that the initial morphology of the precursor is maintained well after pyrolysis. The nanowire morphology was also obtained through the TEM images. In Fig. 2d, e, the HRTEM shows that the nanowire structures consist of MoP cores and graphene shells (Fig. 2d). The nitrogen adsorption/desorption isotherm at 77 K of MoP@PC indicating the specific surface area of 11.7 m^2^ g^−1^ (Fig. S5).

The HRTEM image in Fig. 2e clearly shows the crystal planes with a d-spacing of 0.28 nm, corresponding to the (100) lattice of the MoP nanoparticles [42], further confirming that the MoP was synthesized successfully with a high degree of crystallinity. Moreover, the MoP nanoparticles are surrounded by thick graphite shells with an interlayer distance of 0.34 nm, and the carbon shells number at about 10 layers.

**Fig. 3:**
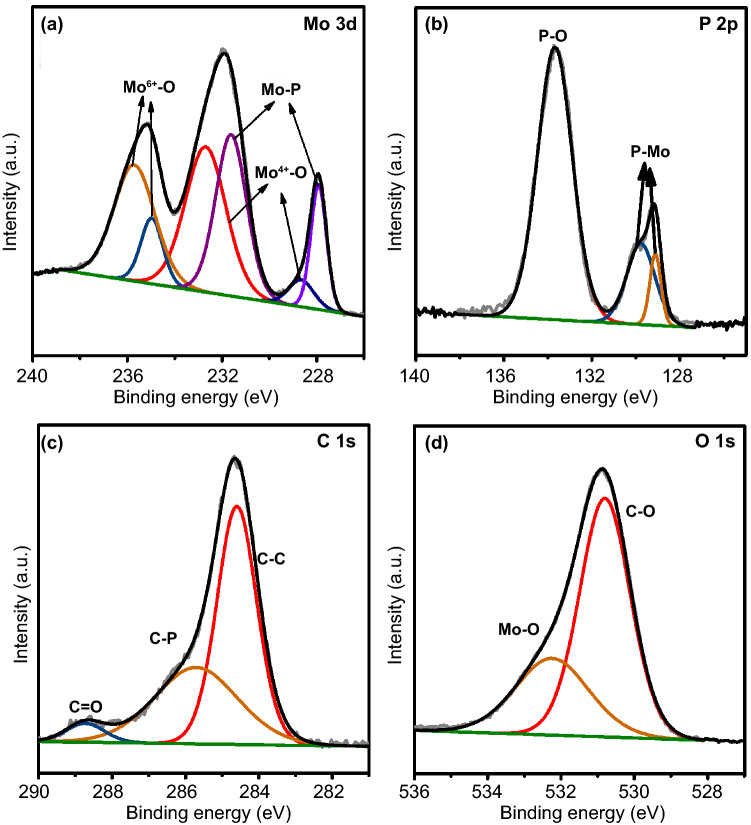
High-resolution XPS of **a** Mo 3d, **b** P 2p, **c** C 1 s, and **d** O 1 s of MoP@PC

To further confirm the chemical compositions of the MoP@PC, Raman spectroscopy was performed (Fig. 2f). The deconvoluted D (≈ 1323 cm^−1^) and D′ (1600 cm^−1^) bands originate from defects such as the edge sites and structural disorders in the graphite shell of MoP@PC, while the observed G band (≈ 1568 cm^−1^) is assigned to the in-plane stretching between *sp*^2^-graphitic carbons. The deconvoluted D_4_ band (≈ 1200 cm^−1^) indicates the presence of amorphous carbon, while the D_3_ band (≈ 1490 cm^−1^) can be ascribed to the disordered graphitic lattice [29]. The low ratio of the G band demonstrates the existence of a highly disordered graphite structure in the carbon shell of MoP@PC [43]. The uniform distribution without significant aggregations of phosphorus, carbon, and molybdenum is observed through TEM energy-dispersive X-ray (EDX) spectroscopy, as shown in Fig. 2g–j. XPS measurements were applied to elucidate the surface chemistry of MoP@PC. The XPS spectra of MoP@PC indicate the existence of Mo, P, C, and O (Fig. S6). Figure 3a–d shows the typical high-resolution XPS profiles of Mo 3d, P 2p, C 1 s, and O 1 s in the MoP@PC, respectively. Two peaks (Fig. 3a) at the low binding energies of 228.3 and 231.5 eV, are assigned to Mo^3+^ in MoP. In Fig. 3a, the four peaks at 236.2, 234.4 (Mo^6+^ 3d_3/2_/3d_5/2_), 233.5, and 229.0 (Mo^4+^ 3d_3/2_/3d_5/2_) eV indicate that a small amount of MoP on the surface was oxidized to MoO_3_ or MoO_2_ [44]. The content of the molybdenum oxide present on the surface of MoP@PC was quite low, so it could not be detected by XRD. The P 2p peaks (Fig. 3b) at 129.5 and 130.4 eV are attributed to low valence P in MoP, and the peak at a higher binding energy (133.8 eV) is assigned to the PO_4_^3−^ or P_2_O_5_ that arises from surface oxidation of P compounds and MoP species [45]. The graphitic carbon is found to be the major species, as reflected in the main peak of the C 1 s spectrum, at 284.6 eV (Fig. 3c). The peak around 286.1 eV is attributed to the carbon in the C–P bonds [46], which is a product of the phosphorization reaction and carbon in the graphene. The peak around 288.5 eV can be attributed to C=O. Figure 3d shows the XPS spectrum of the O 1 s energy region, which can be deconvoluted into two peaks. The molybdenum oxides caused the peak at 532.2 eV, and another peak at 530.2 eV can be attributed to the C–O bonds between the oxygen-containing groups and C atoms.

These results reveal that the one-step annealing of the Mo-MOF precursor in a nitrogen atmosphere and appropriate annealing temperatures can encapsulate MoP in a thick carbon layer. During calcination, the Mo and P in the Mo-MOF precursor form MoP in situ. Simultaneously, the organic moiety in the Mo-MOF structure can provide carbon sources for the in situ generation of a carbon matrix surrounding the MoP. The intrinsic coordination environment of Mo-MOF causes the phosphorus-doped carbon shell to adhere strongly to the MoP core. Taking advantage of the unique metal-support interactions between the MoP and carbon matrix, enhanced electron transport could be achieved, which facilitates the control of the restructured active surface.

### Electrocatalytic Measurement

**Fig. 4:**
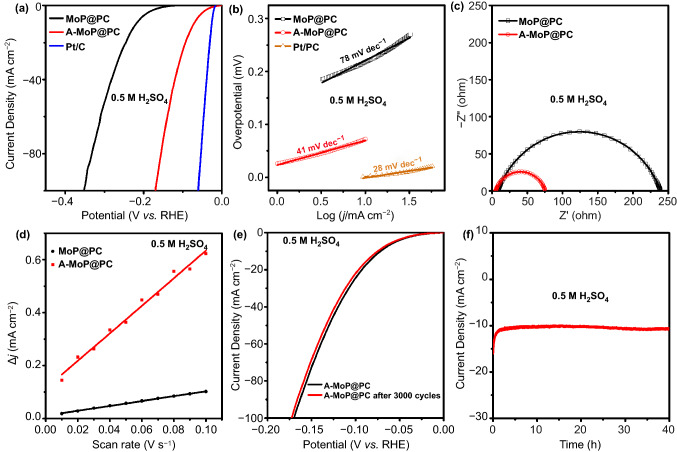
**a** LSV curves, **b** Tafel plots, **c** Nyquist plots, and **d** plots of the capacitive currents of MoP@PC and A-MoP@PC. **e** and **f** Stability test of A-MoP@PC for HER in 0.5 M H_2_SO_4_

The electrocatalytic activity of MoP@PC was investigated in an Ar-saturated 0.5 M H_2_SO_4_ solution. To mitigate the influence of platinum, a carbon rod was used as the counter electrode. The HER performance of the commercial 20 wt% Pt/C catalyst was measured for comparison. The LSV curves are shown in Fig. 4a. The black curve shows the initial performance of MoP@PC. Its high overpotential (*η*_10_ = 233 mV) could be caused by the negative effect of the excessive surface carbon layer shown in TEM results (Fig. 2d, e). Chen et al. [47] eliminated the carbon using oxygen plasma, which requires a plasma generator and does not satisfy the requirements of in situ activation on the electrode. In this study, we found that simple electrochemical activation using CV (a part of the CV activated process is shown in Fig. S7) could restructure the surface with significantly improved HER performance. In comparison with other activation methods, this type of electrochemical activation approach is very simple yet effective for constructing active surfaces. As shown in Fig. 4a, the HER performance of MoP@PC after activation for 12 h (termed as A-MoP@PC) is significantly improved. To achieve a current density of 10 mA cm^−2^, the A-MoP@PC only required an overpotential of 68 mV, which is much lower than that of freshly synthesized MoP@PC, and better than most reported electrocatalysts (Table S1). The Tafel plots of the electrocatalysts were measured to evaluate the intrinsic HER kinetic process. As shown in Fig. 4b, the Tafel slopes of MoP@P and A-MoP@PC were 78 and 41 mV dec^−1^, respectively. The significant decrease in the Tafel slope illustrates that, after 12 h of CV activation, the rate-limiting step for the HER of A-MoP@PC changes from the Volmer step to the Heyrovsky step, which indicates that the Volmer step is accelerated significantly and the reaction kinetics are improved.

The performance improvement of A-MoP@PC in terms of the HER became more apparent based on the Nyquist plots (Fig. 4c) obtained using electrochemical impedance spectroscopy (EIS). The charge transfer resistance of MoP@PC decreased dramatically after CV activation, suggesting that A-MoP@PC has more facile electrode kinetics. The electrochemical active surface areas (ECSA) of MoP@PC and A-MoP@PC were calculated using the double-layer capacitance (C_dl_) [48–50], and could be utilized to evaluate the intrinsic activity [51]. The calculated C_dl_ values (see more details in the supporting information) of MoP@PC and A-MoP@PC (Fig. 4d) are 0.9 and 5.2 mF cm^−2^, respectively. The corresponding ECSA values are 22.9 cm^2^ for MoP@C and 148.7 cm^2^ for A-MoP@C. The increased C_dl_ and ECSA values of A-MoP@PC demonstrated that the higher electrocatalytic activity after activation was caused by the increases of the restructured active surface. In addition to the aforementioned features, stability is an essential factor affecting the performance of electrocatalysts. The stability of the A-MoP@PC catalyst was evaluated using a CV scan with 3000 cycles. As shown in Fig. 4e, A-MoP@PC had similar polarization curves, demonstrating negligible losses in catalytic activity. The chronoamperometric electrolysis curve for 40 h in Fig. 4f also indicates the good durability of A-MoP@PC in acidic electrolytes.

### Underlying Mechanism

**Fig. 5:**
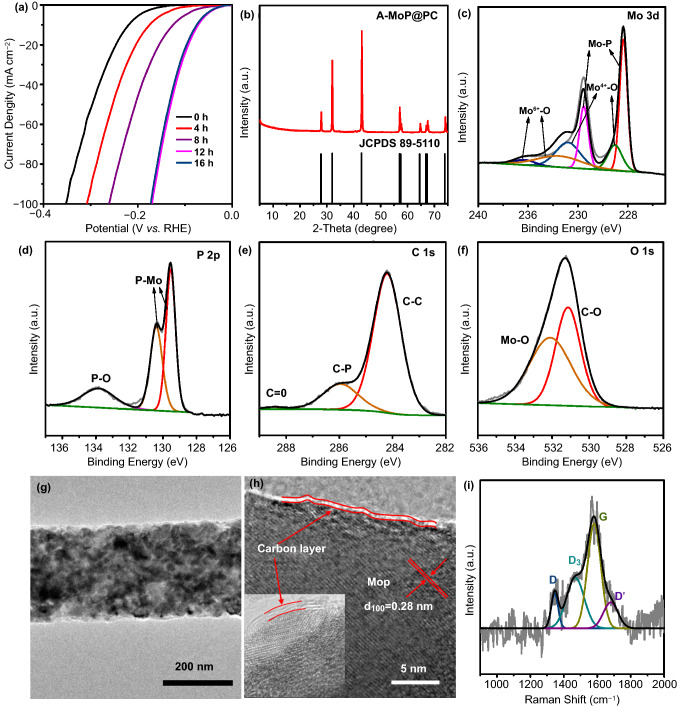
**a** HER polarization curves of MoP@PC activation for different times in 0.5 M H_2_SO_4_. **b** XRD pattern of A-MoP@PC. High-resolution XPS of **c** Mo 3d, **d** P 2p, **e** C 1 s, and **f** O 1 s of A-MoP@PC. **g** TEM and **h** HRTEM of A-MoP@PC. **i** Raman spectrum of A-MoP@PC

It is of great research interest to investigate the CV-induced electrochemical activation mechanism. The HER polarization curves of catalysts after 0, 4, 8, 12, and 16 h of electrochemical activation CV cycles are shown in Fig. 5a. The four curves for 0, 4, 8, and 12 h differ significantly, which indicates an improvement in HER electrocatalytic performance, proving that the accessible potent catalytic surfaces increase gradually from 0 to 12 h. After 12 h of electrochemical activation, the variation trend ceased. The polarization curve of the catalyst after 16 h of electrochemical activation almost overlapped with that for 12 h of electrochemical activation, suggesting that the restructuring process was completed and the restructured structure was stable.

To acquire a deeper understanding of the underlying mechanism, A-MoP@PC was investigated using different types of characterization. To understand the morphology and material composition of the A-MoP@PC catalyst, XRD, XPS, BET, TEM, Raman, and X-ray near-edge structure (XANES) characterizations were performed. The XRD patterns in Fig. 5b show that the bulk phase of A-MoP@PC remains unchanged after electrochemical activation. Figure 5c–f shows the XPS profiles of Mo 3d, P 2p, C 1 s, and O 1 s of A-MoP@PC, respectively. In comparison with the Mo 3d (Fig. 3a) and P 2p (Fig. 3b) profiles of MoP@PC, the stronger peaks of Mo 3d (Fig. 5c) at lower binding energies (227.9 and 231.8 eV) can be assigned to Mo^3+^, and the peak located at 129.8 eV of P 2p (Fig. 5d) proves the improved penetration of MoP for A-MoP@PC, confirming that a portion of the surface carbon layer disappeared. As shown in Fig. 3c, d for MoP@PC, no significant change can be seen in terms of C 1 s (Fig. 5e) and O 1 s (Fig. 5f). As shown in Fig. S9, the A-MoP@PC displays similar N_2_ adsorption/ desorption isotherm with MoP@PC, the specific surface area of A-MoP@PC is 14 m^2^ g^−1^, and there is no significant change in the pore size distribution. The TEM and HRTEM images in Fig. 5g, h, for A-MoP@PC, show that the nanowire structure is maintained well after electrochemical activation, and the surface carbon layers become thinner than the MoP@PC (Fig. 2d, e). Figure 5i shows the Raman spectra of sample A-MoP@PC, which significantly differs from that of MoP@PC, which is shown in Fig. 2f. The disappearance of the D_4_ band demonstrates the dissolution of the amorphous carbon during electrochemical activation. The observed G band (≈1577 cm^−1^) confirms the preservation of stable graphitic carbon on the surface. This could be attributed to the fact that the amorphous carbon shell dissolved in the harsh chemical environment during the electrochemical surface restructuring process. Thick graphite exfoliation takes place continuously until it reaches the closest 1–3 layers to the MoP core; subsequently, the modified electronic structure inhibits further exfoliation.

To assess the structural stability of A-MoP@PC, SEM/TEM was performed. SEM images of the post-HER sample indicated its well-defined nanowire structures, similar to the A-MoP@PC before the HER stability testing (Fig. S10). TEM and HRTEM images (Fig. S11) of A-MoP@PC after the stability testing clearly show that the regular nanowire was well covered by ultrathin carbon layers, demonstrating that the A-MoP@PC still well maintained its original features after the long-term stability test. Surface composition and elements valences of the A-MoP@PC after stability were evaluated by XPS. Results are shown in Fig. S12 with a good preservation of the Mo 3d, P 2p, C 1 s, and O 1 s binding energies. High-resolution XPS spectra for Mo 3d (Fig. S12a), P 2p (Fig. S12b), C 1 s (Fig. S12c), and O 1 s (Fig. S12d) confirmed negligible differences between the original and post-HER samples, suggesting that the sample properly retained its chemical composition and structure after long-term amperometry process.

**Fig. 6:**
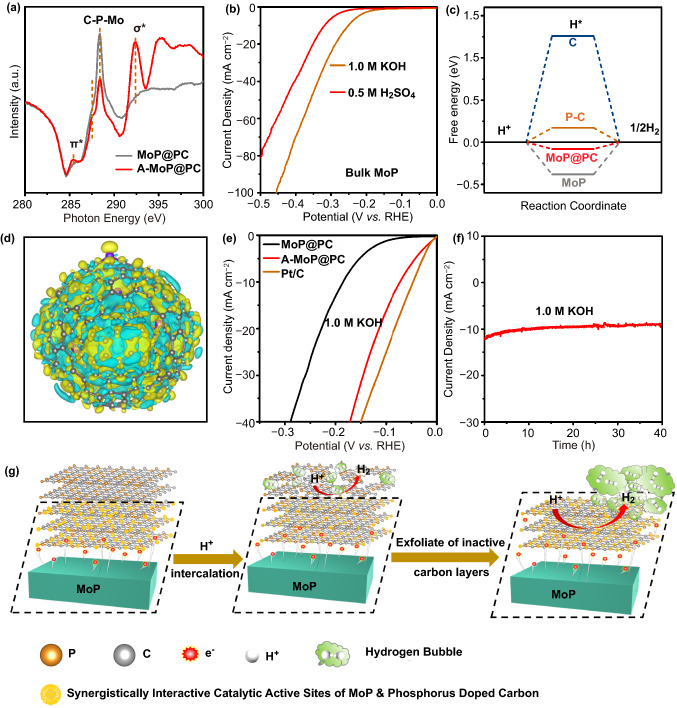
**a** C K-edge XANES of MoP@PC and A-MoP@PC. **b** HER polarization curves of commercial MoP in 0.5 M H_2_SO_4_ and 1.0 M KOH. **c** The calculated free energy diagrams for HER of various catalysts. **d** Three-dimensional charge-density difference for MoP@PC hybrids with an isovalue of 0.0006 e Å^−3^. Yellow and glaucous isosurfaces represent charge accumulation and depletion in the space with respect to isolated MoP clusters and phosphorus-doped carbon shell. **e** HER polarization curves of MoP@PC and A-MoP@PC in 1.0 M KOH. **f** Stability test of A-MoP@PC in 1.0 M KOH. **g** Schematic representation for the restructuring process and mechanism

XANES measurements were carried out to investigate the interaction between the MoP nanoparticles and phosphorus-doped carbon layers. Figure 6a shows the C K-edge XANES spectra of MoP@PC and A-MoP@PC. The absorption features at 285.4 and 292.4 eV can be assigned to the C 1 s transitions of the graphitic C–C π* and C–C δ* states, respectively [52]. For A-MoP@PC, the intensity of the peaks for π* and δ*, increased significantly, indicating that the graphitization degree of A-MoP@PC is improved, which can be attributed to the dissolution of the amorphous carbon; this can be also observed in the aforementioned Raman spectra. There are two peaks between the peak for π* and δ*. The peak located at about 288 eV of A-MoP@PC can be attributed to a graphene analog of the interlayer state in graphite, which indicates the thinning of the thick carbon shell and the creation of the ultrathin graphene layers [53]. The peak at 288.4 eV can be ascribed to Mo–P–C bonds [54–57], suggesting a significant chemical interaction between the MoP and phosphorus-doped carbon layers. The intensity for the Mo–P–C peak of A-MoP@PC is slightly weaker than that of MoP@PC, which could be attributed to the elimination of the superfluous carbon and the thinning of the phosphorus-doped carbon shell during the electrochemical surface restructuring process. This result is also consistent with the measurements discussed above.

To further understand the activation process and identify the nature of the active sites of the catalysts, we investigated the impact of thiocyanate ions (SCN^−^) on the HER activity (Fig. S13). SCN^−^ ions can poison the active metal centers because of their strong binding ability with metal atoms [57]. After the addition of SCN^−^ ions, the Mo active sites of MoP@PC were blocked. As shown in Fig. S13, the polarization curve of the MoP@PC electrocatalyst in the 0.5 M H_2_SO_4_ electrolyte exhibits a marked cathodic shift after poisoning by KSCN (5 mM), implying that the synergistic interaction between MoP and the phosphorus-doped graphene plays a critical role in the catalytic process. Furthermore, MoP also has a certain catalytic effect. To understand the influence of MoP on the catalytic process, the catalytic activity and stability of commercial MoP were further confirmed for comparison. As shown in Fig. 6b, the commercial MoP needed an overpotential of 319 mV to achieve the current density of 10 mA cm^−2^ in 0.5 M H_2_SO_4_. As shown in Fig. S14, when estimated by galvanostatic measurement at a constant current of 10 mA cm^−2^ in 0.5 M H_2_SO_4_, obvious performance attenuation was observed within 10 h, the current density fallen ~ 24% over 15 h. The unsatisfactory activity and poor stability of commercial MoP further highlight the significant synergistic interactions between the phosphorus-doped carbon and MoP.

DFT simulations using the Vienna Ab initio Simulation Package (VASP) of Mede A software were performed to understand the fundamental reaction mechanism of the HER catalytic process, the relevant theoretical models for which are shown in Figs. S15–S18. It has been proven that the adsorption free energy of H (ΔGH*) is an appropriate parameter for use in evaluating the HER activity of a catalyst, and a catalyst that gives ΔGH* ≈ 0 is considered to be a promising candidate for the HER, ensuring rapid proton/electron transfer and a quick hydrogen-release process. As shown in Fig. 6c, the PC exhibits a ΔGH* value of 0.1716 eV, which is much smaller than the value for a carbon shell (1.259 eV), indicating that introducing the P dopant in the carbon layers can enhance the HER activity. Furthermore, the ΔGH* of MoP@PC (-0.079 eV) was comparatively higher than that of the pristine MoP surface (-0.38 eV) and PC, which suggests that the phosphorous dopants and enclosed MoP nanocrystals can synergistically promote the HER performance of graphene shells. In summary, the high HER activity of MoP@PC is attributed to the synergy between the MoP and ultrathin P-doped carbon layer. This is in good agreement with the experimental results. The charge-density difference of model MoP@PC was also calculated and is shown in Fig. 6d. It can be seen that the electrons transferred from the MoP core to the P-doped carbon shell, increasing the electron density in the carbon shell; the synergistic effect between MoP and P-doped carbon can reduce the ΔGH* values to promote the adsorption of H*, enhancing the electrocatalytic performance of the HER.

A schematic of the restructuring process and its underlying mechanism is shown in Fig. 6g. It can be seen that the intrinsic coordination environment of the MOF precursor causes the phosphorus-doped carbon shell of the MoP@PC to adhere strongly to the MoP core. Owing to the strong electronic liberation ability, the MoP core with sufficient electrons can serve as electron donors. The electrons shuttle across the phosphorus-doped graphene layers in proximity (1–3 layers) on the MoP core, modifying the surface electronic structures and charge distributions [58], changing the electronic properties of the inert phosphorus-doped graphene layer significantly. This results in alterations to the binding energies of the reaction intermediates on the phosphorus-doped graphene surface, enhancing the electrocatalytic activity. Subsequently, the superfluous inert carbon shell could gradually be stripped off during the CV process owing to mechanical erosion caused by the evolved hydrogen bubbles; therefore, only the highly active graphene surface remained. Because the modified electronic properties by the MoP core could strengthen the adherence of phosphorus-doped graphene shells, the shutting electrons allow the MoP to adhere strongly to the thin carbon layer; therefore, the binding force between the polarized phosphorus-doped graphene shell and MoP core is strengthened. Further, when the negatively charged carbon layers were modified by MoP, the HER was more likely to take place on the outmost surface, where the reaction molecules and media would be triggered to decompose as soon as they contacted the external high-activity graphene surface. The surface species do not change any more, which indicated that the further surface reconstruction terminated. The phosphorus-doped graphene shells can stabilize the MoP cores under harsh conditions, and the redistributed electrons at the interface of the MoP cores and surface carbon layers enhance the HER activity. Therefore, the catalytic activity of A-MoP@PC is significantly higher than that of any single component. The positive perturbation of the electron distribution endows the outermost surface with excellent catalytic activity, further catalyzing the HER. The synergistic interaction significantly boosts the electrocatalytic activity and stability.

To extend the applicability of this method, the HER performance of A-MoP@PC was investigated in an alkaline electrolyte (1.0 M KOH); a commercial Pt/C sample was evaluated for comparison. Figure 6e shows the LSV curves of MoP@PC and A-MoP@PC catalysts in a 1.0 M KOH solution. The A-MoP@PC exhibited a small overpotential, of 67 mV for *η*_*1*0_, which is better than most reported nonprecious electrocatalysts under alkaline conditions. The corresponding Tafel plots are shown in Fig. S19. The Tafel slopes of MoP@PC and A-MoP@PC are 99 and 40 mV dec^−1^, respectively, exhibiting more efficient HER kinetics for an A-MoP@PC catalyst with the Heyrovsky mechanism. The catalytic activity and stability of commercial MoP in 1.0 M KOH were performed as contrasts. The overpotential was as high as 252 mV to obtain the current density of 10 mA cm^−2^ (Fig. 6b). Meanwhile, the stability of commercial MoP was also poor, the current density decayed rapidly as time progressed (Fig. S14b). The excellent HER catalytic activity of A-MoP@PC is close to that of the Pt/C catalyst and better than most non-noble-metal-based catalysts (Table S1). The chronoamperometric electrolysis curve in Fig. 6f also exhibits the high durability of A-MoP@PC in alkaline electrolytes; the current density shows ignored decline within 40 h.

## Conclusions

Herein, a new class of phosphorus-doped carbon-coated molybdenum phosphide (A-MoP@PC) electrocatalysts were synthesized through the pyrolysis of a Mo-MOF precursor, followed by a simple electrochemical cycling treatment. The in situ transformation of metal nodes/organic ligands in MOF led to the highly dispersed MoP being protected by phosphorus-doped carbon. During the electrochemical process, the electrons of the inner MoP flow through the carbon layers to the outer surface owing to the greater electron-escape capability, extending the range of MoP in terms of adhering to the thin carbon layer. As a result, the superfluous inactive carbon shell can be stripped off gradually by the CV process, leading to the restructuring of the electrocatalysts when the active surface carbon was exposed. The activated carbon surfaces not only enhance the catalytic activity but also simultaneously strengthen the durability of the catalyst. DFT calculations indicate that the superior HER performance is a result of the modulation of the electron density and the electronic potential distribution at the graphene surface caused by penetrating electrons from the MoP core. The encapsulated MoP cores act like hearts pumping out electrons into the carbon shell; the electron-rich characteristics of the polarized carbon layer contribute to an increase in the active sites and significantly reduce ΔGH*. These advantages of the electronic modulated structure provide the low overpotentials of 68 mV and 67 mV for the HER, allowing it to reach a current density of 10 mA cm^−2^ in 0.5 M H_2_SO_4_ and 1.0 M KOH, respectively. This strategy can help design superior HER electrocatalysts, providing a promising approach for developing durable electrochemical catalysts.

## Supplementary Information

Below is the link to the electronic supplementary material.

Supplementary file1 (PDF 1710 kb)
