# Supplementary material for: Electrochemical Surface Restructuring of Phosphorus-Doped Carbon@MoP Electrocatalysts for Hydrogen Evolution
Source: Nanomicro Lett. 2021 Oct 21;13:215. doi: 10.1007/s40820-021-00737-w (PMC8531175; doi:10.1007/s40820-021-00737-w)
Supplement: Supplementary file 1 — Supplementary file1 (PDF 1710 kb) [file 40820_2021_737_MOESM1_ESM.pdf]

Supporting Information for

Electrochemical Surface Restructuring of Phosphorus Doped Carbon@MoP Electrocatalysts for Hydrogen Evolution

Huimin Jiang[1, 3], Liting Yan[1, *], Shuo Zhang[3], Yanchao Zhao[3], Xue Yang[1], Yameng Wang[1], Jianxing Shen[1], Xuebo Zhao[1, 3*], Lianzhou Wang[2, *]

[1]School of Materials Science and Engineering, Qilu University of Technology (Shandong Academy of Sciences), No.3501, Daxue Road, Changqing District, Jinan, 250353, P. R. China

[2]Nanomaterials Centre, School of Chemical Engineering and Australian Institute for Bioengineering and Nanotechnology, The University of Queensland, St Lucia, QLD 4072, Australia

[3]College of Chemical Engineering, China University of Petroleum (East China), No. 66, West Changjiang Road, Huangdao District, Qingdao, 266580, P. R. China

*Corresponding authors. E-mail: yanlt@qlu.edu.cn (Liting Yan); zhaoxuebo@upc.edu.cn (Xuebo Zhao); l.wang@uq.edu.au (Lianzhou Wang)

S1 Experimental Section

S1.1 Chemicals

Sodium molybdate(VI) dihydrate ($Na_2MoO_4·2H_2O$), alpha, alpha'-Dibromo-p-xylene (97 wt%), paraformaldehyde (96 wt%), potassium hydroxide (KOH, ≥85.0 wt%), sulfuric acid (95.0～98.0%), hydrochloric acid (36.0~38.0 wt%) and ethanol (≥ 99.7 wt%) were purchased from Sinopharm Chemical Reagent Co. Ltd. Hydrogen bromide (33 wt% in acetic acid) and commercial molybdenum phosphide (99.5%) were purchased from Aladdin. Carbon rod was purchased from Shandong Haike Chemical Group Co., Nafion solution (5 wt% in a mixture of lower aliphatic alcohols and water) and platinum on carbon (Pt/C, 10 wt%) were purchased from Sigma-Aldrich Co. LLC. All reagents were used without further purification.

S1.2 Material Synthesis

S1.2.1 Synthesis of p-xylylenediphosphonic acid (H_4xdp) [S1]*:*

The ligand was synthesized by reacting alpha, alpha'-Dibromo-p-xylene with triethyl phosphite and followed by refluxing the obtained oil with conc. hydrochloric acid according to the literature method. Block colorless crystals were obtained from the water solution by slow evaporation.

S1.2.2 Synthesis of $[(MoO_2)_2(xdp)(H_2O)_2]·2H_2O$ [S1]

Mo-MOF precursor was prepared according to previous work [S1]. In a typical procedure, $Na_2MoO_4·2H_2O$ (0.240 g, 1.0 mmol) was stirred together with p-xylylenediphosphonic acid (H_4xdp) (0.140 g, 0.5 mmol) in 16ml deionised water. The pH of the solution was adjusted to pH 1 by dropwise addition of conc. hydrochloric acid. The acidified solution was then placed in a 25 cm^3 Ace pressure tube and heated at 120 °C for 15 h. The resultant white crystalline material was thoroughly washed with deionised water several times and dried at 80 °C for 12 h under vacuum.

S1.2.3 Preparation of MoP@PC Nanowires

In a typical procedure, 500 mg Mo-MOF precursor was placed in a porcelain boat. Then, the boat was heated at 900 °C under a constant flow of N_2 at 30 mL min^{-1} for 120 min with the warming rate of 20 °C min^{-1}. The final black powder was collected when the temperature dropped to room temperature under N_2.

S1.3 Electrochemical Activation

The in-situ electrochemical activation was carried out in 0.5 M H_2SO_4 under a N_2 atmosphere to avoid possible oxidation caused by O_2 in air. This was conducted by using the three-electrode system of CHI 760E electrochemical workstation (CH Instruments, Inc., Shanghai). MoP@PC was used as the working electrode, carbon rod was used as the counter electrode, Ag/AgCl (saturated KCl-filled) was used as the reference electrode. The electrochemical activation was performed by cycle voltammetry (CV) from -0.2 to 0.2 V vs RHE in 0.5M H_2SO_4, portion of the activation is shown in **Fig. S7**.

S1.4 Characterization

The crystal structure of sample was characterized by powder X-ray diffraction (XRD) (PANalytical Inc.) using Cu Kα irradiation operating at 45 KV and 40 mA with a fixed slit. Morphology of sample was observed by a JEOL JSM-7500F (Japan) Field Emission Scanning Electron Microscopy (FESEM). TEM (HRTEM) images were measured using a JEOL JEM2100F (Japan)Transmission Electron Microscope for investigating the information on lattice and fringe. Nitrogen sorption isotherms were measured at 77 K using an Autosorb volumetric gas sorption analyzer (Quantachrome, USA). TGA was conducted on a thermal analyzer (Mettler Toledo TGA/SDTA85, Canada) from room temperature to 1000 °C in N_2 atmosphere. X-ray photoelectron spectroscopy (XPS) analyses were performed with a Thermo ESCALAB 250 (USA) spectrometer using an Al K_α (1486.6 eV) photon source. Raman spectrum was recorded using JY HR800 under ambient conditions. The X-ray absorption near edge structure (XANES) measurement was performed at Singapore Synchrotron Light Source, facility for catalysis research (XAFCA) beamline.

Electrochemical measurements were performed at room temperature, catalyst ink was typically made by dispersing 20 mg of catalyst in 2 mL ethanol. After adding 0.5 mL of 0.05 wt% of Nafion solution (Gashub, Singapore) and ultrasonication, an aliquot of 5 µL was pipetted onto the glassy carbon electrode (0.0706 cm^2) to reach the catalyst loading of 0.56 mg cm^{-2}. In a three-electrode configuration, Polarization curves were collected by CHI 760E electrochemical workstation at room temperature. Carbon rod as the counter electrode, Ag/AgCl and saturated calomel electrode (SCE) were used as the reference electrodes in acid and alkaline electrolyte, respectively. All the potentials shown were recorded with respect to the reversible hydrogen electrode (RHE) without IR correction. Current density was normalized to the geometrical area of the working electrode. Polarization data are collected at the scan rate of 5 mV s^{-1} on a rotation disk electrode under 2000 rpm. EISs were carried out in a potentiostatic mode in the frequency range of 10^6 to 1 Hz with the amplitude of 5 mV.

S1.5 Electrochemically Active Surface Area

The electrochemically capacitance measurements were conducted by cyclic voltammograms from 0.10 to 0.30 V with various scan rates (10, 20, 30, 40, 50, 60, 70, 80, 90, 100 mV s^{-1}) as shown in **Fig. S5**. The capacitive currents were measured in a potential where no faradic processes were observed. According to the previous report [2], the specific capacitance, a flat standard with 1 cm^2 of real surface area, is approximately 40 µF cm^{-2}. Thus, the electrochemical active surface area can be calculated by following Eq. (S1):

$$A_{ECSA}=\frac{\text{electrochemical capacitance}}{40\ \text{Mf cm}^{-2}\ \text{per cm}^2_{ECSA}} \quad (S1)$$

S1.6 Calculated Electrochemically Active Surface Area

MoP@PC:

$$\text{MoP@PC} = \frac{0.9\ \text{mF cm}^{-2}}{40\ \mu\text{F cm}^{-2}\ \text{per cm}^2_{\text{ECSA}}} = 22.9\ \text{cm}^2 \quad \text{(S2)}$$

A-MoP@PC:

$$\text{A-MoP@PC} = \frac{5.2\ \text{mF cm}^{-2}}{40\ \mu\text{F cm}^{-2}\ \text{per cm}^2_{\text{ECSA}}} = 148.7\ \text{cm}^2 \quad \text{(S3)}$$

S1.7 DFT Calculations

All calculations were performed using Vienna Ab-initio Simulation Package (VASP) of MedeA software, the generalized gradient approximation (GGA) of Perdew−Becke−Ernzerhof (PBE) is used for the exchange-correlation functional [S3-S5] The MoP@C_{240} model was built by encapsulating a MoP cluster with a graphitic carbon cage C_{240}, which performed well in previous study [S6, S7]. In the construction of model MoP@$C_{239}P_1$, and $C_{239}P_1$, P atom was introduced by substituting C atom in the carbon cage. All structures were fully relaxed to the ground state and spin-polarization was considered in all calculations. The convergence of energy and forces were set to 1×10^{-4} eV and 0.01 eV Å^{-1}, respectively. An energy cutoff of 400 eV and a Gamma k-point sampling were found to get convergent geometry. For HER, the free energies of the intermediates were obtained by $\Delta G(\text{H*}) = \Delta E(\text{H*}) + \Delta ZPE - \text{T}\Delta S$, where $\Delta E(\text{H*})$, ΔZPE and ΔS is the binding energy, zero-point energy change and entropy change of adsorption H, respectively. The ΔZPE and ΔS were obtained according to the method reported by Norskov [S8, S9].

The adsorption energy (E_{ads}) is given by

$$E_{\text{ads}} = E_{\text{adsorbed slab + adsorbate}} - (E_{\text{adsorbed slab}} + E_{\text{adsorbate}})$$

where $E_{\text{adsorbed slab + adsorbate}}$, $E_{\text{adsorbed slab}}$, and $E_{\text{adsorbate}}$ correspond to the total energy of the optimized system, the adsorbed slab, and the isolated adsorbate molecule, respectively.

S2 Supplementary Figures and Tables

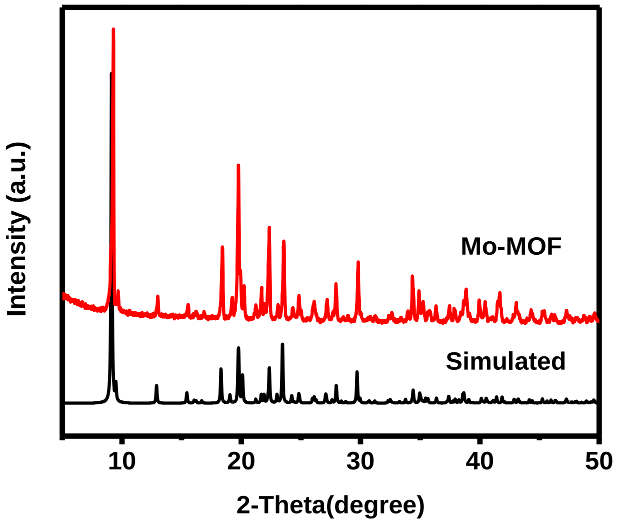

Fig. S1 XRD patterns of Mo-MOF

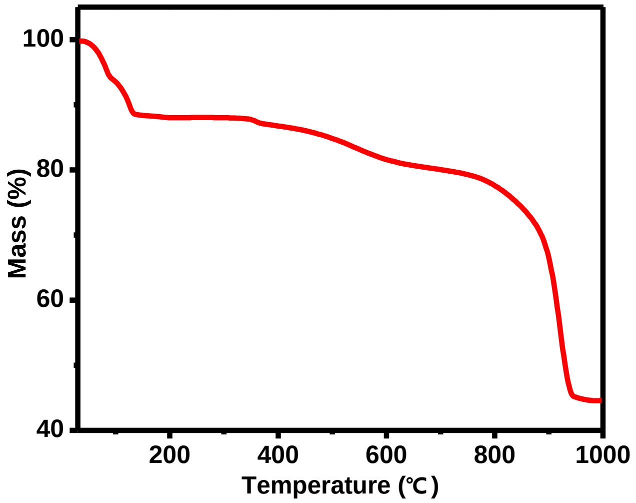

Fig. S2 TGA of Mo-MOF precursor in N_2 atmosphere

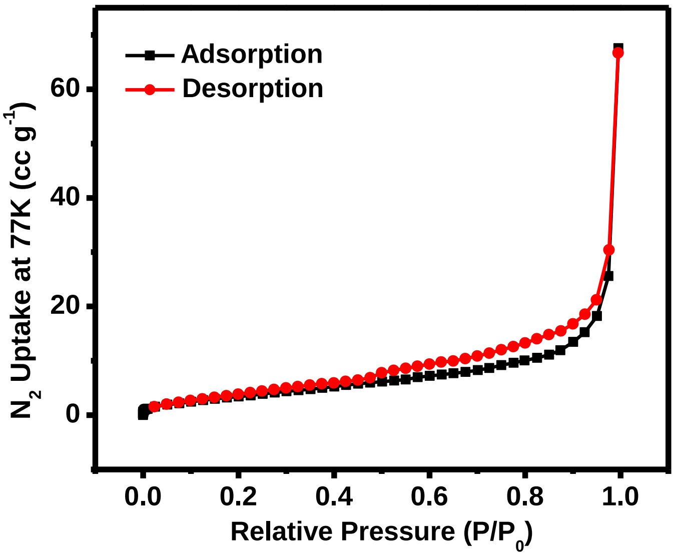

Fig. S3 N_2 adsorption/desorption isotherm at 77 K of Mo-MOF

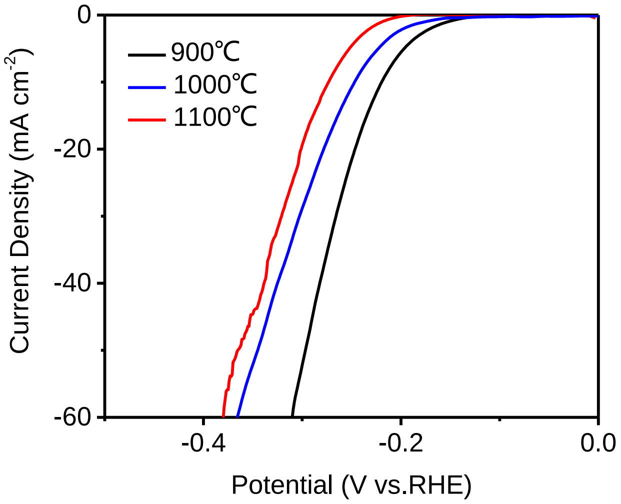

Fig. S4 LSV curves of MoP calcined at 900-1100 ℃ in 0.5 M H_2SO_4

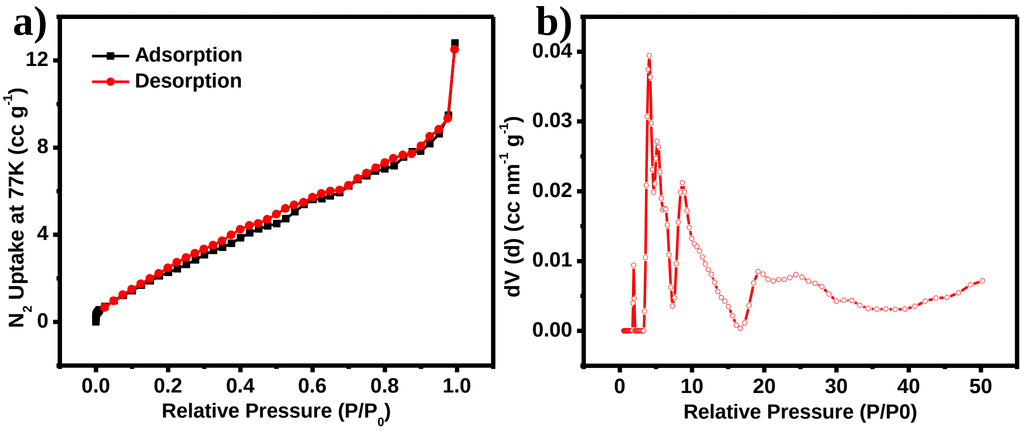

Fig. S5 a) N_2 adsorption/desorption isotherm at 77 K and b) corresponding NLDFT pore diameter distribution of MoP@PC

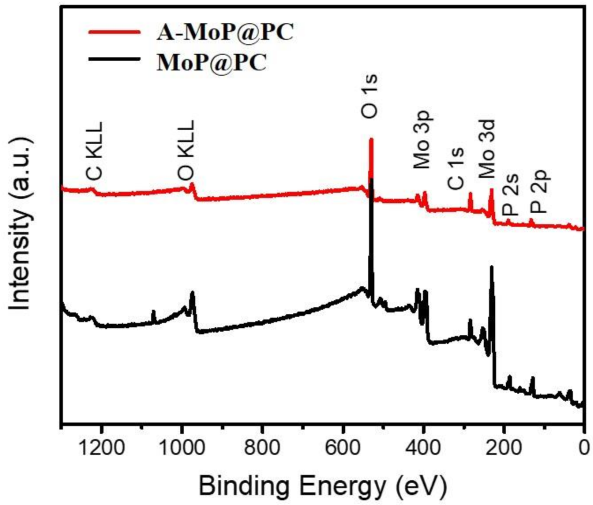

Fig. S6 XPS spectrum of MoP@PC and A-MoP@PC

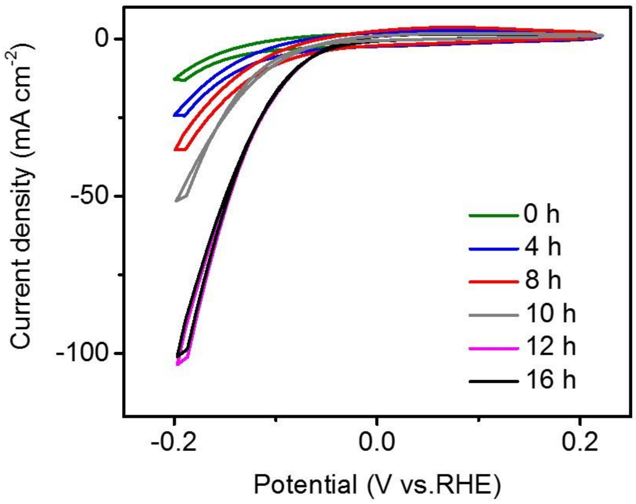

Fig. S7 CV curves of A-MoP@PC activation for different time

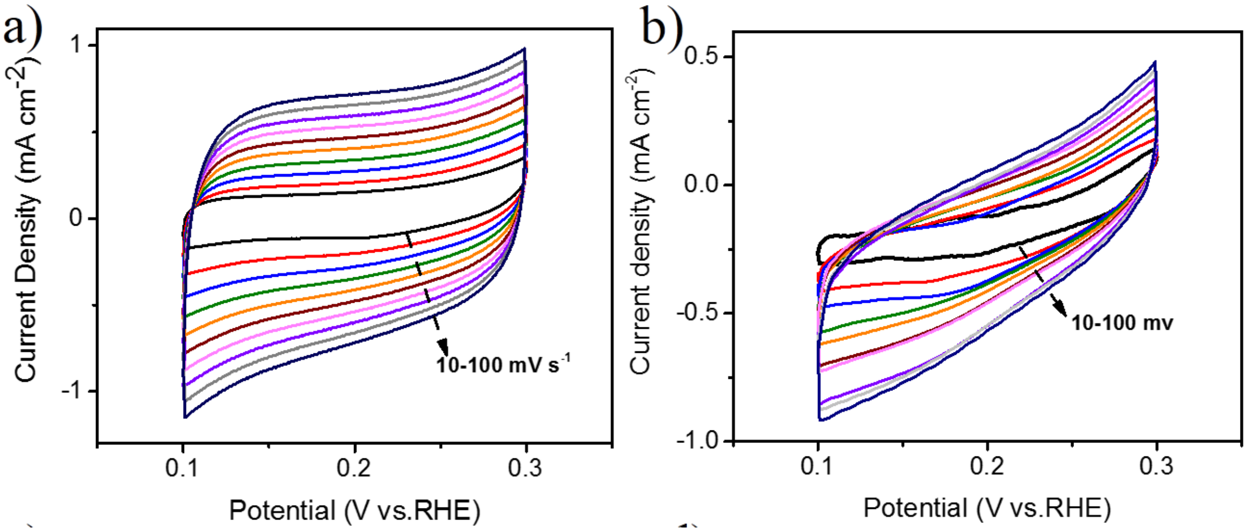

Fig. S8 Cyclic voltammograms of **a**) MoP@PC and **b**) A-MoP@PC after activation with various scan rates

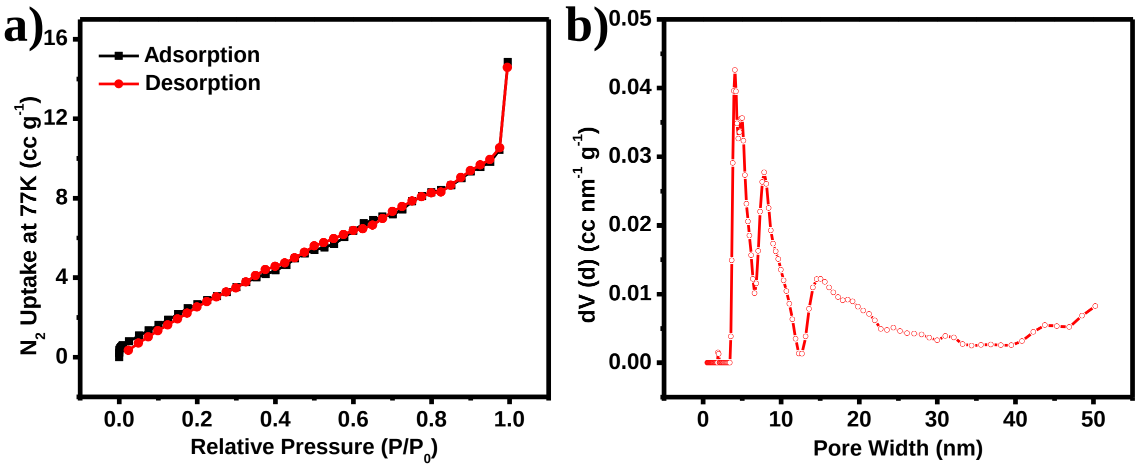

Fig. S9 **a**) N_2 adsorption/desorption isotherm at 77 K and **b**) corresponding NLDFT pore diameter distribution of A-MoP@PC

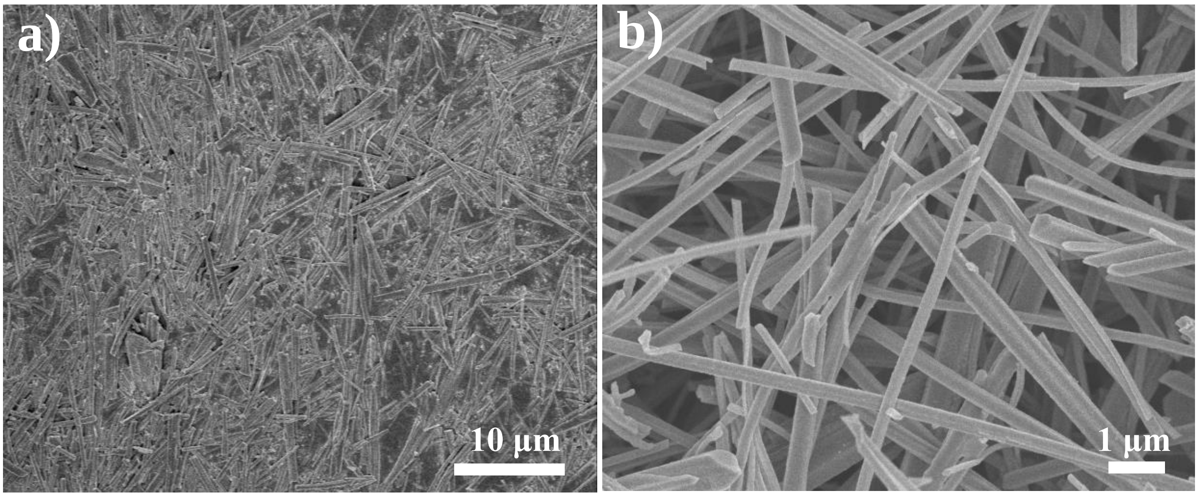

Fig. S10 **a**) and **b**) SEM image of A-MoP@PC after the stability test

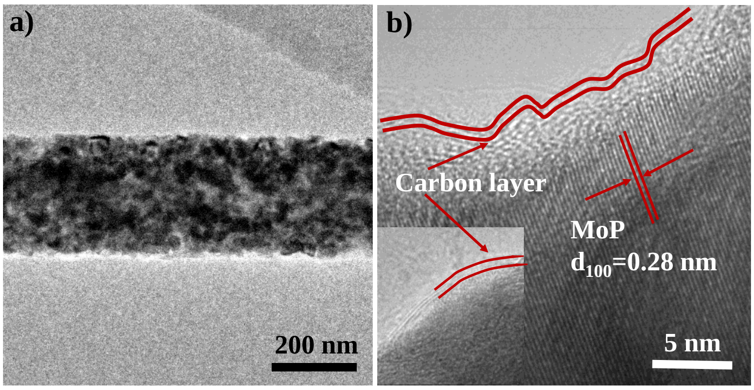

Fig. S11 a) TEM and **b**) HRTEM of A-MoP@PC after the stability test

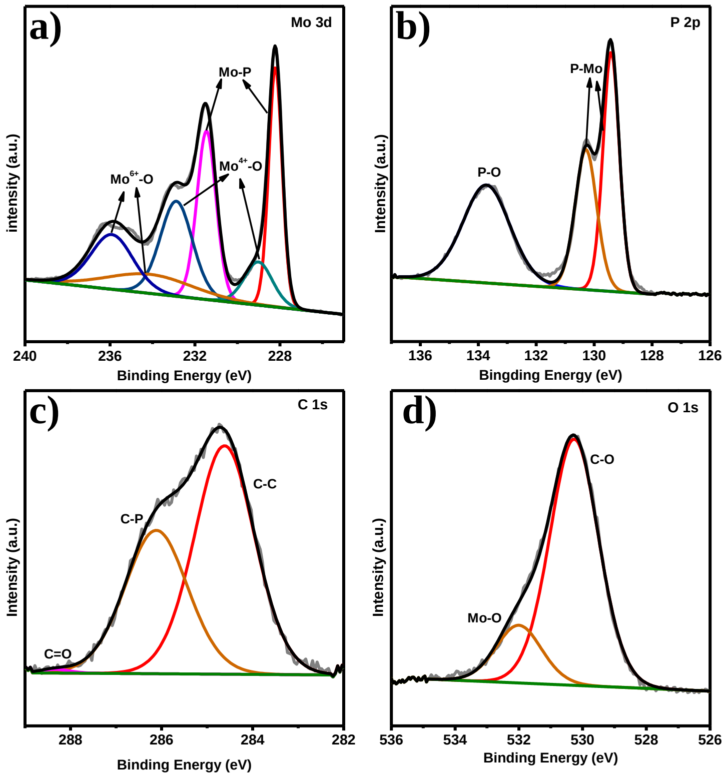

Fig. S12 High resolution XPS of **a**) Mo 3d, **b**) P 2p, **c**) C 1s and **d**) O 1s of A-MoP@PC after the stability test

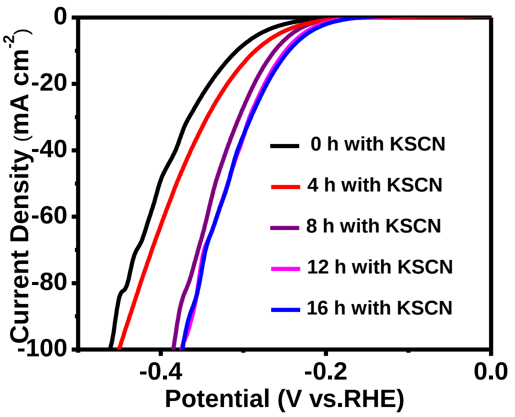

Fig. S13 HER polarization curves of MoP@PC activation for different time after addition of 5 mM SCN^- ions in 0.5 M H_2SO_4

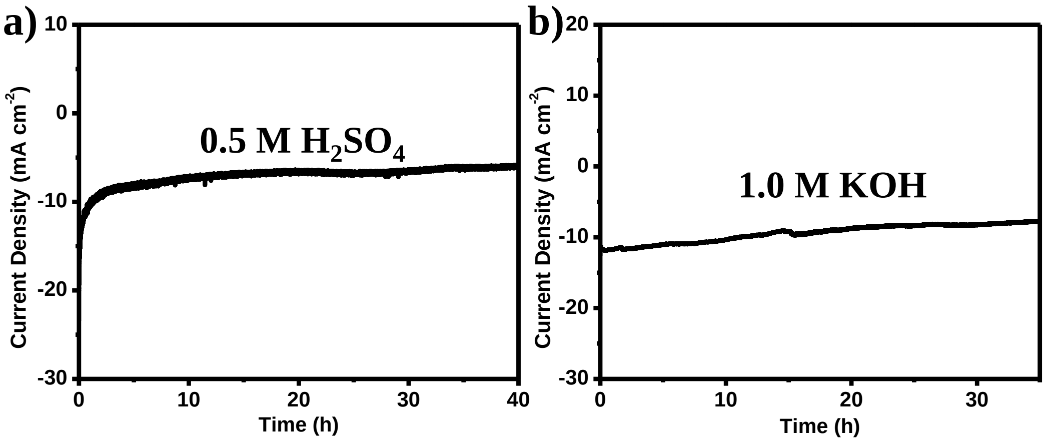

Fig. S14 Chronoamperometric stability test of commericial MoP for HER in **a**) 0.5 M H_2SO_4 and **b**) 1.0 M KOH

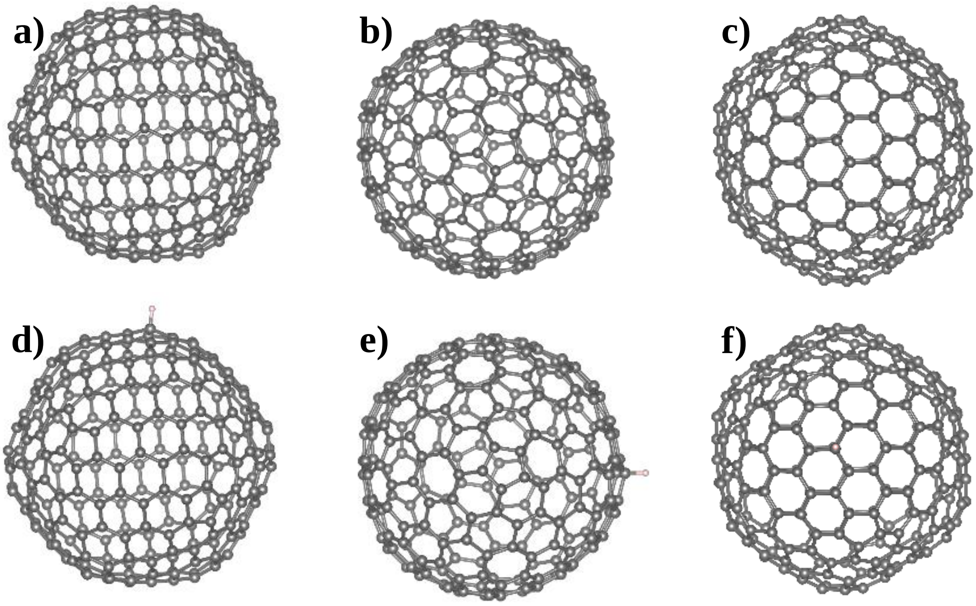

Fig. S15 a-c Computational models of C. **d-f** Configurations of adsorbates of structures on C for HER

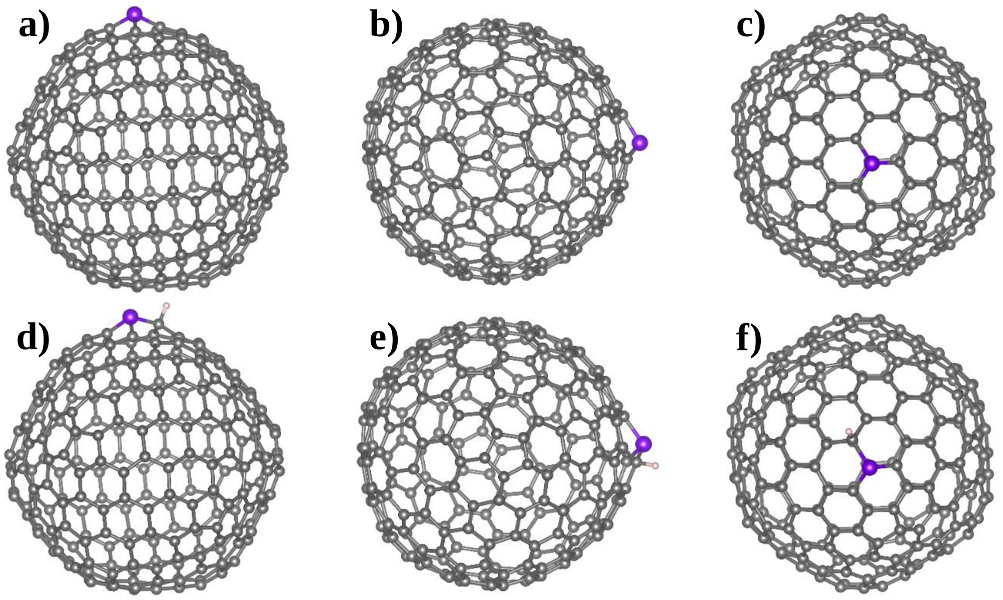

Fig. S16 a-c Computational models of PC. **d-f** Configurations of adsorbates of structures on PC for HER

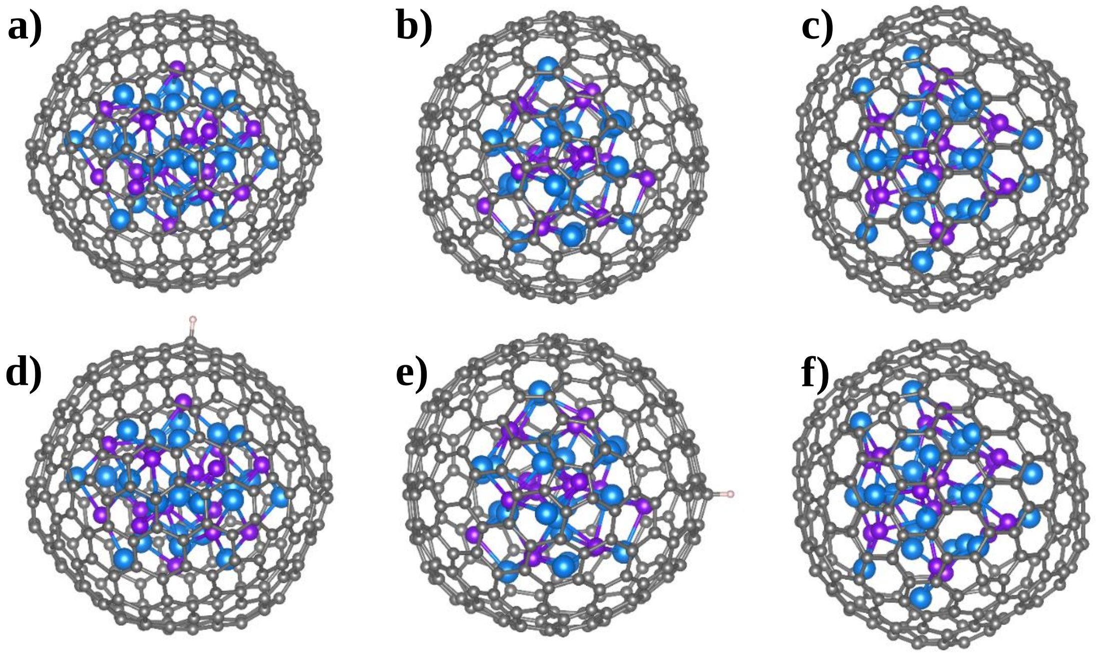

Fig. S17 a-c Computational models of A-MoP@C. **d-f** Configurations of adsorbates of structures on A-MoP@C for HER

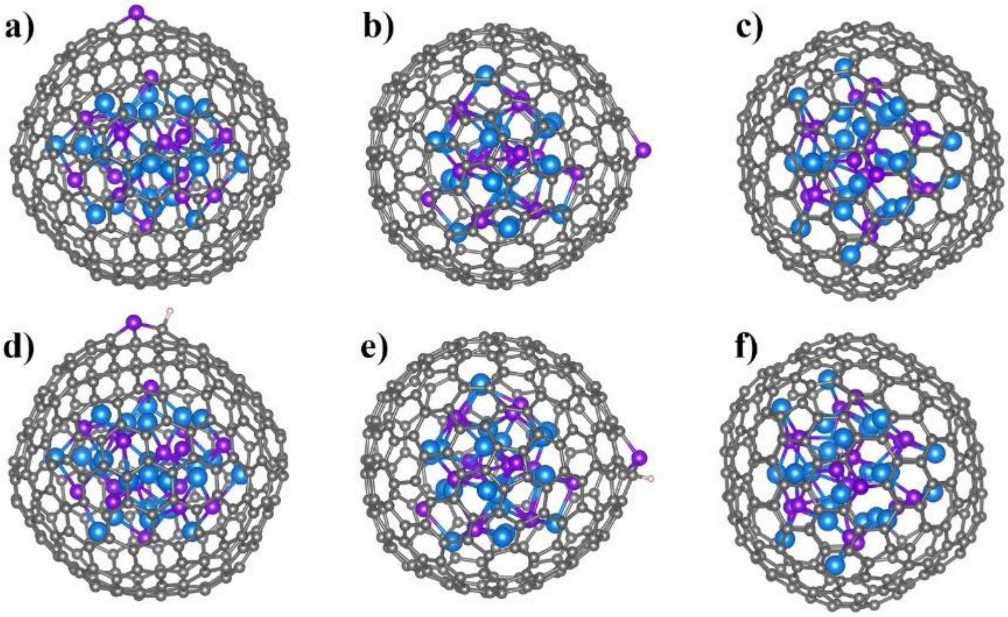

Fig. S18 a-c Computational models of A-MoP@PC. **d-f** Configurations of adsorbates of structures on A-MoP@PC for HER

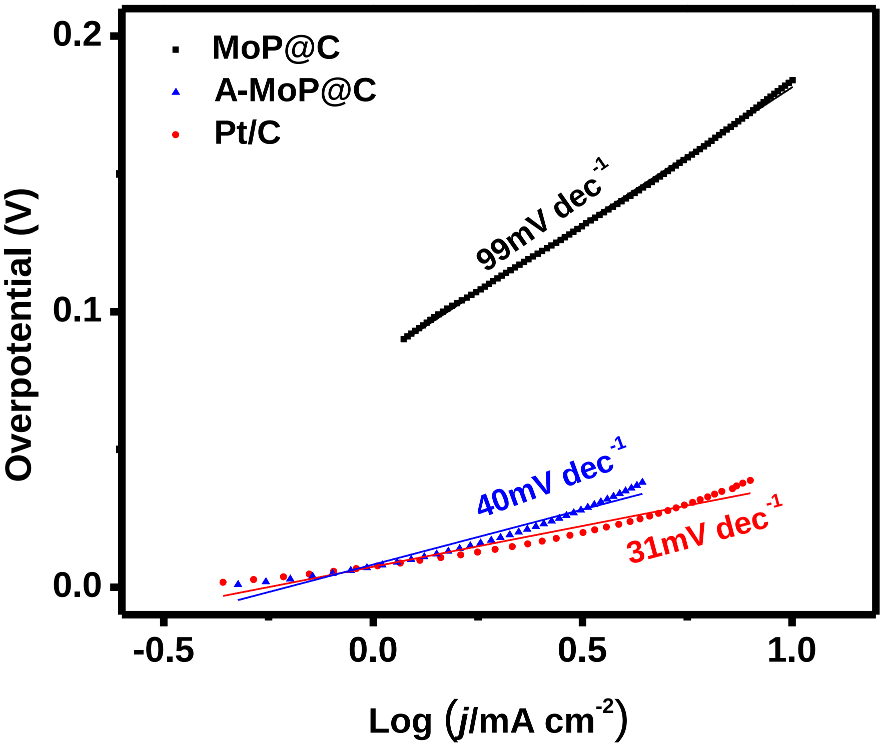

Fig. S19 Tafel plots of MoP@PC, A-MoP@PC and Pt/C in 1.0 M KOH

Table S1 Comparison of HER activity for A-MoP@PC and recently reported noble metal-free hydrogen evolution catalysts

Catalyst	η_{10} (mV)	Tafel slope (mV dec^{-1})	Electrolyte	Refs.
N@MoPC$_x$	108	69.4	0.5 M H_2SO_4	[S2]
MoS_2-Me-10%	136	37	0.5 M H_2SO_4	[S10]
WS_2	137	54	0.5 M H_2SO_4	[S11]
N-MoS_2/CN	114	46.8	0.5 M H_2SO_4	[S12]
Cu3P@NPPC	89	76	0.5 M H_2SO_4	[S13]
meso-Fe-MoS_2/$CoMo_2S_4$	122	90	1.0 M KOH	[S14]
O-CoP	98	59.9	1.0 M KOH	[S15]
Fe-N_4 SAs/NPC	202	123	1.0 M KOH	[S16]
NiCoFe@C	260	105	1.0 M KOH	[S17]
Mn-doped NiS_2/Ni foam	71	57	1.0 M KOH	[S18]
MoP@NCHSs	92	62	1.0 M KOH	[S19]
0.02Ni–MoP	102<br>162	58.1<br>102.6	0.5 M H_2SO_4<br>1.0 M KOH	[S20]
Fe_3C-Co/NC	298<br>238	100.3<br>108.8	0.5 M H_2SO_4<br>1.0 M KOH	[S21]
CoP/NiCoP NTs	125<br>133	71<br>88	0.5 M H_2SO_4<br>1.0 M KOH	[S22]
np-η-MoC NSs	122<br>119	53<br>39	0.5 M H_2SO_4<br>1.0 M KOH	[S23]
MoP@NPSC	71<br>50	75<br>45	0.5 M H_2SO_4<br>1.0 M KOH	[S24]
Ti-MoP	93.6	44.5	0.5 M H_2SO_4	[S25]
MoP/CDs	70	77.49	1.0 M KOH	[S26]
P-MoP/Mo_2N	89<br>89	53<br>78	0.5 M H_2SO_4<br>1.0 M KOH	[S27]
N-MoP-800	175<br>125	69<br>69	0.5 M H_2SO_4<br>1.0 M KOH	[S28]
Ni_2P/MoP-CC	290<br>78	63<br>64	0.5 M H_2SO_4<br>1.0 M KOH	[S29]
**A-MoP@PC**	**68**<br>**67**	**41**<br>**40**	**0.5 M H_2SO_4**<br>**1.0 M KOH**	**This work**

Supplementary References

[S1] A.A. Ayi, A.D. Burrows, M.F. Mahon, V.M. Sebestyen, A molybdenum diphosphonate network structure exhibiting reversible dehydration and selective uptake of methanol. CrystEngComm **15**, 9301 (2013). https://doi.org/10.1039/c3ce40484f

[S2] Y. Huang, J. Ge, J. Hu, J. Zhang, J. Hao et al., Nitrogen-doped porous molybdenum carbide and phosphide hybrids on a carbon matrix as highly effective electrocatalysts for the hydrogen evolution reaction. Adv. Energy Mater. **8**, 1701601 (2018). https://doi.org/10.1002/aenm.201701601

[S3] I.J.P. Perdew, K. Burke, M. Ernzerhof, Generalized gradient approximation made simple. Phys. Rev. Lett. **77**, 3865 (1996). https://doi.org/10.1103/PhysRevLett.77.3865

[S4] I.G. Kresse, D. Joubert, From ultrasoft pseudopotentials to the projector augmented-wave method. Phys. Rev. B **59**, 1758 (1999). https://doi.org/10.1103/PhysRevB.59.1758

[S5] I.K. Hoshino, F. Shimojo, Ab initio molecular dynamics for expanded and compressed liquid alkali metals. J. Phys.: Condens. Matter **8**, 9315 (1996). https://doi.org/10.1088/0953-8984/8/47/022

[S6] I.J. Su, Y. Yang, G. Xia, J. Chen, P. Jiang et al., Ruthenium-cobalt nanoalloys encapsulated in nitrogen-doped graphene as active electrocatalysts for producing hydrogen in alkaline media. Nat. Commun. **8**, 14969 (2017). https://doi.org/10.1038/ncomms14969

[S7] I.X. Cui, P. Ren, D. Deng, J. Deng, X. Bao, Single layer graphene encapsulating non-precious metals as high-performance electrocatalysts for water oxidation. Energy Environ. Sci. **9**, 123 (2016). https://doi.org/10.1039/C5EE03316K

[S8] I.H. Zhang, Z. Ma, J. Duan, H. Liu, G. Liu et al., Active sites implanted carbon cages in core–shell architecture: highly active and durable electrocatalyst for hydrogen evolution reaction. ACS Nano **10**, 684 (2015). https://doi.org/10.1021/acsnano.5b05728

[S9] I. J. K. Norskov, T. Bligaard, A. Logadottir, J. Kitchin, J.G. Chen et al., Trends in the exchange current for hydrogen evolution. J. Electrochem. Soc. **152**, J23 (2005). https://doi.org/10.1149/1.1856988

[S10] I.H. Kwak, I.S. Kwon, T.T. Debela, J. Seo, J.P. Ahn et al., Two-dimensional MoS_2–melamine hybrid nanostructures for enhanced catalytic hydrogen evolution reaction. J. Mater. Chem. A **7**, 22571 (2019). https://doi.org/10.1039/C9TA07802A

[S11] M. Wang, L. Zhang, M. Huang, Q. Zhang, X. Zhao et al., One-step synthesis of a hierarchical self-supported WS_2 film for efficient electrocatalytic hydrogen evolution. J. Mater. Chem. A **7**, 22405 (2019). https://doi.org/10.1039/C9TA07868A

[S12] H. Wang, X. Xiao, S. Liu, C.L. Chiang, X. Kuai et al., Structural and electronic optimization of MoS_2 edges for hydrogen evolution. J. Am. Chem. Soc. **141**, 18578 (2019). https://doi.org/10.1021/jacs.9b09932

[S13] R. Wang, X.Y. Dong, J. Du, J.Y. Zhao and S.Q. Zang, MOF-derived bifunctional Cu_3P nanoparticles coated by a N,P-codoped carbon shell for hydrogen evolution and oxygen reduction. Adv. Mater. **30**, 1703711(2018). https://doi.org/10.1002/adma.201703711

[S14] Y. Guo, J. Tang, J. Henzie, B. Jiang, W. Xia et al., Mesoporous iron-doped $MoS_2/CoMo_2S_4$ heterostructures through organic–metal cooperative interactions on spherical micelles for electrochemical water splitting. ACS Nano **14**, 4141 (2020). https://doi.org/10.1021/acsnano.9b08904

[S15] Z. Liu, J. Ai, M. Sun, F. Han, Z. Li et al., Phosphorous-doped graphite layers with outstanding electrocatalytic activities for the oxygen and hydrogen evolution reactions in water electrolysis. Adv. Funct. Mater. **30**, 1910741 (2020). https://doi.org/10.1002/adfm.201910741

[S16] Y. Pan, S. Liu, K. Sun, X. Chen, B. Wang et al., A bimetallic Zn/Fe polyphthalocyanine-derived single-atom $Fe-N_4$ catalytic site:a superior trifunctional catalyst for overall water splitting and Zn–air batteries. Angew. Chem. Int. Ed. **57**, 8614 (2018). https://doi.org/10.1002/anie.201804349

[S17] X. Lu, X. Tan, Q. Zhang, R. Daiyan, J. Pan et al., Versatile electrocatalytic processes realized by Ni, Co and Fe alloyed core coordinated carbon shells. J. Mater. Chem. A **7**, 12154 (2019). https://doi.org/10.1039/C9TA01723B

[S18] L. Zeng, Z. Liu, K. Sun, Y. Chen, J. Zhao et al., Multiple modulations of pyrite nickel sulfides via metal heteroatom doping engineering for boosting alkaline and neutral hydrogen evolution. J. Mater. Chem. A **7**, 25628 (2019) https://doi.org/10.1039/C9TA08030A

[S19] D. Zhao, K. Sun, W.C. Cheong, L. Zheng, C. Zhang et al., Ultra-tuning of the aperture size in stiffened ZIF-8_Cm frameworks with mixed-linker strategy for enhanced CO_2/CH_4 separation. Angew. Chem. Int. Ed. **58**, 2 (2019). https://doi.org/10.1002/anie.201813331

[S20] W. Xiao, L. Zhang, D. Bukhvalov, Z. Chen, Z. Zou et al., Hierarchical ultrathin carbon encapsulating transition metal doped MoP electrocatalysts for efficient and pH-universal hydrogen evolution reaction. Nano Energy **70**, 104445 (2020). https://doi.org/10.1016/j.nanoen.2020.104445

[S21] C.C. Yang, S.F. Zai, Y.T. Zhou, L. Du and Q. Jiang, Fe_3C-Co nanoparticles encapsulated in a hierarchical structure of N-doped carbon as a multifunctional electrocatalyst for ORR, OER, and HER. Adv. Funct. Mater. **25**, 1901949 (2019). https://doi.org/10.1002/adfm.201901949

[S22] Y. Lin, K. Sun, S. Liu, X. Chen, Y. Cheng et al., Construction of CoP/NiCoP nanotadpoles heterojunction interface for wide pH hydrogen evolution electrocatalysis and supercapacitor. Adv. Energy Mater. **9**, 1901213 (2019). https://doi.org/10.1002/aenm.201901213

[S23] C. Tang, H. Zhang, K. Xu, Q. Zhang, J. Liu et al., Unconventional molybdenum carbide phases with high electrocatalytic activity for hydrogen evolution reaction. J. Mater. Chem. A **7**, 18030 (2019) https://doi.org/10.1039/C9TA04374H

[S24] Y. Jiao, H. Yan, R. Wang, X. Wang, X. Zhang et al., Porous plate-like MoP assembly as an efficient pH-universal hydrogen evolution electrocatalyst. ACS Appl. Mater. Interfaces **12**, 49596 (2020). https://doi.org/10.1021/acsami.0c13533

[S25] I. Jang, K. Im, H. Shin, K.-S. Lee, H. Kim et al., Electron-deficient titanium single-atom electrocatalyst for stable and efficient hydrogen production. Nano Energy **78**, 105151 (2020). https://doi.org/10.1016/j.nanoen.2020.105151

[S26] H. Song, Y. Li, L. Shang, Z. Tang, T. Zhang et al., Designed controllable nitrogen-doped carbon-dots-loaded MoP nanoparticles for boosting hydrogen evolution reaction in alkaline medium. Nano Energy **72**, 104730 (2020). https://doi.org/10.1016/j.nanoen.2020.104730

[S27] Y. Gu, A. Wu, Y. Jiao, H. Zheng, X. Wang et al., Two-dimensional porous molybdenum phosphide/nitride heterojunction nanosheets for pH-universal hydrogen evolution reaction. Angew. Chem. Int. Ed. **60**, 6673 (2021). https://doi.org/10.1002/anie.202016102

[S28] Y. Li, N.P. Nidamanuri, A. Jiang, Z. Wang, Q. Li et al., In situ construction of tandem nitrogen-doped MoP nanocrystals for high-efficient electrocatalytic hydrogen evolution. Electrochimica Acta **342**, 136059 (2020). https://doi.org/10.1016/j.electacta.2020.136059

[S29] Y. Xu, M. Yan, Z. Liu, J. Wang, Z. Zhai et al., Nanostructures Ni_2P/MoP @ N – doping porous carbon for efficient hydrogen evolution over a broad pH range. Electrochimica Acta **363**, 137151 (2020). https://doi.org/10.1016/j.electacta.2020.137151
